# Sequence Variations of Full-Length Hepatitis B Virus Genomes in Chinese Patients with HBsAg-Negative Hepatitis B Infection

**DOI:** 10.1371/journal.pone.0099028

**Published:** 2014-06-05

**Authors:** Fung-Yu Huang, Danny Ka-Ho Wong, Wai-Kay Seto, An-Ye Zhang, Cheuk-Kwong Lee, Che-Kit Lin, James Fung, Ching-Lung Lai, Man-Fung Yuen

**Affiliations:** 1 Department of Medicine, The University of Hong Kong, Queen Mary Hospital, Hong Kong, China; 2 State Key Laboratory for Liver Research, The University of Hong Kong, Queen Mary Hospital, Hong Kong, China; 3 Hong Kong Red Cross Blood Transfusion Service, Hospital Authority, Hong Kong, China; University of Pretoria/NHLS TAD, South Africa

## Abstract

**Background:**

The underlying mechanism of HBsAg-negative hepatitis B virus (HBV) infection is notoriously difficult to elucidate because of the extremely low DNA levels which define the condition. We used a highly efficient amplification method to overcome this obstacle and achieved our aim which was to identify specific mutations or sequence variations associated with this entity.

**Methods:**

A total of 185 sera and 60 liver biopsies from HBsAg-negative, HBV DNA-positive subjects or known chronic hepatitis B (CHB) subjects with HBsAg seroclearance were amplified by rolling circle amplification followed by full-length HBV genome sequencing. Eleven HBsAg-positive CHB subjects were included as controls. The effects of pivotal mutations identified on regulatory regions on promoter activities were analyzed.

**Results:**

22 and 11 full-length HBV genomes were amplified from HBsAg-negative and control subjects respectively. HBV genotype C was the dominant strain. A higher mutation frequency was observed in HBsAg-negative subjects than controls, irrespective of genotype. The nucleotide diversity over the entire HBV genome was significantly higher in HBsAg-negative subjects compared with controls (p* = *0.008) and compared with 49 reference sequences from CHB patients (p* = *0.025). In addition, HBsAg-negative subjects had significantly higher amino acid substitutions in the four viral genes than controls (all p<0.001). Many mutations were uniquely found in HBsAg-negative subjects, including deletions in promoter regions (13.6%), abolishment of pre-S2/S start codon (18.2%), disruption of pre-S2/S mRNA splicing site (4.5%), nucleotide duplications (9.1%), and missense mutations in “α” determinant region, contributing to defects in HBsAg production.

**Conclusions:**

These data suggest an accumulation of multiple mutations constraining viral transcriptional activities contribute to HBsAg-negativity in HBV infection.

## Introduction

Hepatitis B virus (HBV) infection leads to a wide spectrum of liver injuries, ranging from acute self-limiting infection and fulminant hepatitis to chronic hepatitis, liver cirrhosis and hepatocellular carcinoma (HCC) [1]. The viral genome contains four partially overlapping open reading frames (ORFs) (pre-S/S, pre-C/C, P and X) [2]. HBV infection is usually diagnosed when the circulating HBV surface antigen (HBsAg) is detected. Spontaneous loss of HBsAg is a rare event in chronic hepatitis B (CHB) infection [3]–[6]. Loss of serum HBsAg is also observed in patients receiving treatment for CHB with interferon and nucleoside/nucleotide analogues [7], [8].

Although the continued presence of detectable HBsAg in serum is a prominent feature of HBsAg-positive CHB infection or overt CHB infection, HBsAg seroclearance may not signify eradication of HBV. The introduction of sensitive HBV DNA detection tests has revealed the existence of HBsAg-negative HBV infection. This new entity of HBV infection is defined as “the presence of viral DNA in the liver (with detectable or undetectable HBV DNA in the serum) of individuals testing negative for the HBsAg” [9], [10]. Some of these subjects may be people with or without prior medical history of HBV infection can develop to the occult phase [11]. All these scenarios are collectively termed occult hepatitis B infection (OBI) [10].

Clinical evidence reveals that there is generally no reduction in the risk for HCC in CHB patients with HBsAg seroclearance compared with those who are persistently positive for HBsAg [12]–[14]. The virological features and the mechanisms leading to OBI are still unclear, although viral mutations may be one of the significant factors. Many attempts have been done to analyze HBV genome sequences amplified from OBI patients. Most studies focused on searching for mutations on a fragment of HBV genome (mainly the region coding for HBsAg). This is due to the extremely low viral DNA levels in blood or liver tissue in these patients rendering amplification difficult. Mutations have been found in various regions of the HBV genome and may be associated with OBI: (1) mutations in the surface protein affecting antigen detection [15]–[17]; (2) deletions in the pre-S1 region that impair HBV packaging [18], [19]; (3) various mutations in viral regulatory regions that cause decreased in HBsAg expression and/or viral replication [20], [21]; and (4) mutations affecting post-translational protein production [20], [22]. In addition to these potential mechanisms, epigenetic modifications of the HBV genome are also suggested to play a significant role in OBI [23]. However, these studies mainly evaluated viral coding sequences, in particular, the different surface proteins. Owing to the complex organization of viral genome and also the efficient regulation, it is important to study mutations in both gene coding regions as well as the regulatory regions. It remains elusive as to which mutations and variations can lead to OBI. Thus, comprehensive whole genome analyses are still needed.

In the present study, we adopted a highly efficient amplification method, rolling circle amplification (RCA) to amplify full-length HBV genome from sera and liver biopsies of OBI subjects. We primarily aimed at analyzing sequence variations of complete HBV genome in subjects with OBI. The secondary aim was to analyze the effect of pivotal mutations identified on regulatory region(s) on promoter activities of the viral genes.

## Materials and Methods

### Patients and Samples

A total of 185 serum samples (156 from CHB patients with HBsAg seroclearance; 29 from HBsAg-negative patients with detectable HBV DNA at the first presentation) and 60 liver biopsies (21 from CHB patients with HBsAg seroclearance; 39 from HBsAg-negative patients with detectable HBV DNA at the first presentation) were recruited. Another 11 serum samples were collected from HBsAg-positive treatment-naïve CHB patients as controls. These subjects were first identified by testing their blood samples for HBsAg (Abbott Prism, Abbott Laboratories, Abbott Park IL) and by the nucleic acid amplification test (NAT) for HBV DNA (Procleix Trigis system, Novartis Diagnostics, Emeryville, CA; 95% detection limit, 12.2 IU/ml). Serum HBV DNA levels were measured using the COBAS TaqMan HBV Monitor Test (Roche Diagnostics, Branchburg, NJ), with a lower limit of detection of 20 IU/ml (100 copies/ml). HBV DNA levels in the liver biopsies were measured by real-time PCR using the Artus HBV RG assay (Qiagen), which has a linear range of detection of 1.1 IU/ml to >4×10^9^ IU/ml. The present study was approved by the Institutional Review Board, the University of Hong Kong, Hong Kong. Written informed consent was obtained from all patients.

### Rolling Circle Amplification and Sequencing of Full-length HBV Genome

RCA, a powerful PCR-based technique for the amplification of low viral load circular DNA, has been previously described [24]. Briefly, HBV plus-strand DNA inside the virions in the serum was completed using the endogenous HBV polymerase before extraction using the QIAamp MinElute Virus Vacuum Kit (Qiagen). Nicks in the HBV DNA strands were then completed by ligase (Epicentre, Madison, WI). For the liver biopsy samples, total DNA was extracted using the QIAamp Allprep Kit (Qiagen). The HBV DNA samples were subjected to RCA using eight primers spanning the whole HBV genome (Table S1) [24]. Single genome-length HBV DNA was either retrieved directly by *Spe*I digestion (New England Biolabs) or further amplified using the Expand High Fidelity PCR system (Roche, Mannheim, Germany) [25]. This amplified HBV DNA was then purified and sequenced using sequencing primers that cover the whole viral genome (Table S1). Sensitivity and conditions for RCA reaction were tested against samples with known copies of viral DNA (quantified by the Artus real-time PCR kit; Qiagen).

### Nucleotide Sequence and Amino Acid Analysis

Full-length HBV sequences were assembled using the CLC Main Workbench 6.6.2 (CLC Bio, Katrinebjerg, Denmark). HBV genotype was determined using the NCBI genotyping tool and phylogenetic analysis. HBV sequences from OBI subjects were compared with either sequences from the control subjects or genotype-matched HBV sequences of Chinese CHB patients retrieved from the GenBank [26]. Analysis of nucleotide and amino acid diversity (d), as well as phylogenetic analysis, was performed using the MEGA version 5 software [27]. The program SimPlot [28] was used to search for evidence of recombination. Amino acid mutation frequency was defined as the number of amino acid variations per total number of amino acid residues within a particular genomic region. Prediction of RNA secondary structure was performed using the MFOLD software [29].

### In-vitro Analysis on the Effects of Genetic Mutations on Specific Regulatory Regions on HBV Promoter Activities

HBV promoter regions from selected OBI cases and wilde-type controls were amplified using primers containing *Kpn*I and *Nhe*I restriction sites. The *Kpn*I- and *Nhe*I-digested PCR products, containing different mutated HBV promoter regions, were cloned into the reporter vector pGL3-Basic. Positive constructs were confirmed and co-transfected with pRL-TK *Renilla* reporter plasmid into Huh-7 cells using Lipofectamine 2000 (Invitrogen). After 24 hours of incubation, the cells were assayed for luciferase activity using the Dual-Luciferase Reporter Assay System (Promega, Madison, WI). Promoter activities were expressed as a ratio of firefly luciferase to *Renilla* luciferase luminescence. Results were expressed in arbitrary units (AU).

### Statistical Analyses

Differences between categorical variables were analyzed using the Fisher’s exact test or Chi-square test. For continuous variables, the Student’s t-test was used. All statistical analysis was done using GraphPad Prism 5.0 (GraphPad Software, Inc. San Diego, CA). Data are expressed as percentage or mean ± SD. A p-value of <0.05 was considered to be statistically significant.

## Results

### Rolling Circle Amplification for HBV Genomes from OBI

**Figure 1:**
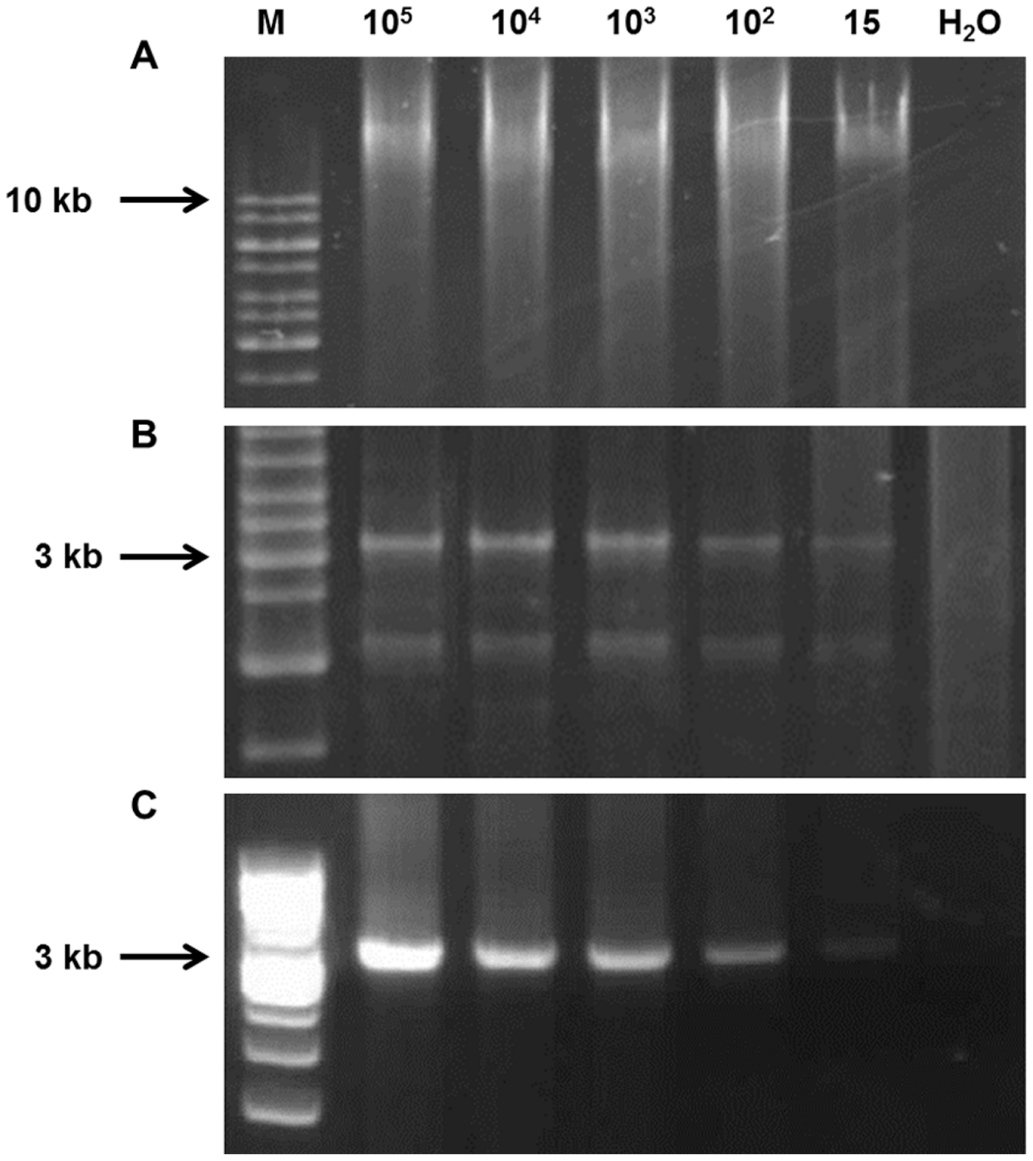
Rolling circle amplification (RCA) of full-length HBV genome. The number of initial HBV DNA template present in each RCA reaction is shown at the top. M, molecular weight marker; H2O, negative control. (A) The high-molecular-weight raw RCA products containing multiple copies of the initial HBV template. (B) The full-length HBV genomes recovered by restriction enzyme, *Spe*I digestion. (C) Full-length HBV genome amplified by using the RCA products as PCR template.

By using known copies of HBV DNA isolated from CHB patients, we demonstrated RCA could amplify down to 15 copies/reaction (Figure 1). Full-length HBV genomes were successfully amplified from 18/60 (30%) liver biopsies and 4/185 (2.2%) serum samples from OBI subjects using this RCA method. Complete HBV genomes were also amplified from all 11 controls. Seventeen out of these 18 OBI subjects had detectable intrahepatic HBV DNA levels (median: 4.07 copies/cell; range: 0.07–15.34 copies/cell). HBV viral load in the 4 serum samples were all under the detection limit of COBAS assay of 20 IU/ml. Full-length HBV genomes were also amplified from 11 HBsAg-positive controls (median: 3.51×10^5^ IU/ml; range: 100–7.34×10^7^ IU/ml).

### Demographic Data and Phylogenetic Analysis

Demographic data of the study subjects are summarized in Table 1. Genotype C was dominant in both OBI group (17/22; 77.3%; 11 with subtype C1, 4 with subtype C2 and 2 outliers) and controls (9/11; 81.8%; all subtype C1). Genotype B was detected in the remaining OBI subjects (5/22; 22.7%; all subtype B2) and controls (2/11; 18.2%; 1 with subtype B2 and 1 outlier) (Figure S1). Evidence of intergenotypic recombination between genotypes B/C was detected in these 3 outliers but only constituted less than 20% of the major parental genotype. The full-length HBV genomes and multiple sub-genomic regions reproduce the same genotyping results, these 3 outliers (2 occult samples and 1 control) were clustered into genotype C and B respectively. Of note, phylogenetic analysis based on the full-length HBV sequences or the 4 individual ORFs cannot distinctly separate OBI from CHB controls (data not shown).

**Table 1:** Clinical characteristics of the study subjects with full-length HBV genome amplified by rolling circle amplification (RCA).

	OBI group	Control group	*P*-value
	(n = 22)	(n = 11)	
Age			
Mean ± SEM	49.18±6.16	54.91±16.54	0.156
Gender			
Male : Female	13∶ 9	5∶6	0.488
HBV genotype (%)			
B	5 (22.7%)	2 (18.2%)	
C	17 (77.3%)	9 (81.8%)	0.763
Mutation frequency in the HBV key regulatory elements			
Enhancer I	17 (77.3%)	6 (54.5%)	0.240
Enhancer II	7 (31.8%)	3 (27.3%)	1.000
DR 1	1 (4.5%)	0 (0%)	n.a.
DR 2	0 (0%)	0 (0%)	n.a.
Pre-S1 promoter	11 (50.0%)	7 (63.6%)	0.712
Pre-S2 promoter	14 (63.6%)	6 (54.5%)	0.714
Core Promoter	22 (100%)	10 (91.0%)	0.333
X Promoter	12 (54.5%)	5 (45.5%)	0.721

(OBI, occult hepatitis B infection; n.a.: not applicable; SEM: standard error of the mean; DR: direct repeat).

### Comparison of HBV Genomic Diversity between OBI and Control Subjects

The nucleotide and amino acid diversity were evaluated with respective to their genotypes. The nucleotide diversity over the entire HBV genome with genotype C was significantly higher in the 17 subjects with OBI when compared with the 9 control subjects (diversity, d = 0.040±0.002 vs. 0.026±0.002, p = 0.008). The nucleotide diversity in these 17 OBI subjects was also significantly higher than that in 49 genotype C reference sequences retrieved from Chinese CHB patients [26] (d = 0.04±0.002 vs. d = 0.030±0.001, p = 0.025). There was no significant difference in the nucleotide diversity between OBI and control subjects with genotype B (d = 0.021±0.002 vs. d = 0.016±0.002, p = 0.279), which may be related to limited number of tested subjects (n = 5 and n = 2, respectively). Similarly, the nucleotide diversity between OBI subjects and 5 genotype B reference sequences retrieved from Chinese CHB patients showed no significant difference (d = 0.016±0.002 vs. d = 0.015±0.002, p = 0.792) [30] (Table 2). In terms of amino acid diversity, there was no significant difference between OBI and control sequences for both genotypes (data not shown).

**Table 2:** Comparison of the nucleotide (nt.) diversity (± SEM) on the HBV genome of occult hepatitis B and control subjects.

HBV regions	Genotype B			Genotype C		
	OBI	Control	P-value	OBI	Control	P-value
Pre-S/S	0.008±0.002	0.006±0.002	0.490	0.030±0.003	0.018±0.003	0.069
Pre-S1	0.009±0.003	0.007±0.003	0.833	0.053±0.007	0.022±0.005	***0.048*** *
Pre-S2	0.011±0.005	0.002±0.002	0.320	0.032±0.008	0.024±0.009	0.603
S	0.007±0.002	0.006±0.002	0.706	0.020±0.003	0.015±0.003	0.461
Pre-C/Core	0.028±0.005	0.009±0.003	***0.045*** *	0.041±0.004	0.024±0.004	0.059
Pre-C	0.017±0.010	0.018±0.011	0.924	0.043±0.013	0.014±0.008	***0.047*** *
Core	0.030±0.005	0.008±0.003	***0.031*** *	0.041±0.004	0.025±0.004	0.099
P	0.013±0.002	0.014±0.002	0.911	0.039±0.003	0.026±0.002	***0.032*** *
X	0.016±0.004	0.036±0.006	0.090	0.027±0.004	0.018±0.004	0.267
Pre-S1 promoter	0.027±0.005	0.011±0.003	0.110	0.055±0.006	0.034±0.005	0.097
Pre-S2/S promoter	0.010±0.004	0.008±0.003	0.684	0.052±0.007	0.020±0.005	***0.037*** *
Full-length HBV	0.021±0.003	0.016±0.002	0.279	0.040±0.002	0.026±0.002	0.008*
Δ Full-length HBV	0.016±0.002	0.015±0.002	0.792	0.040±0.002	0.030±0.002	0.025*

Δ Full-length HBV: OBI sequence compared with 5 genotype B and 49 genotype C reference sequence obtained from Chinese patients with chronic HBV infection. *A statistical difference with p<0.05.

Further analysis on the nucleotide and amino acid diversity on the 4 individual HBV ORFs indicated OBI cases presented slightly higher nucleotide and amino acid diversity than control cases (Table 2). For genotype C cases, the nucleotide diversity in the pre-S1, pre-C and P ORFs was significantly higher in OBI than control cases (p* = *0.048, p* = *0.047 and p* = *0.032, respectively). For genotype B cases, OBI subjects had a significantly higher nucleotide diversity in the pre-C/C and C regions (p = 0.045 and p* = *0.031, respectively) (Table 2), and in the RNase H region (p = 0.049) of the P ORF than control subjects (data not shown). Furthermore, the difference in the nucleotide diversity between OBI and control groups with genotype C in both pre-S/S and pre-C/C regions also showed a similar trend (p = 0.069 and p = 0.059, respectively) (Table 2). There was no significant difference on the amino acid diversity between OBI and control sequences on the 4 ORFs in both genotypes (data not shown). In summary, OBI cases had a higher nucleotide diversity than control cases, and these changes were scattered over the entire viral genome and may not lead to coding changes. This was evidenced by the comparable amino acid diversity between the two groups.

### Mutational Analysis on the pre-S/S Coding Region

**Figure 2:**
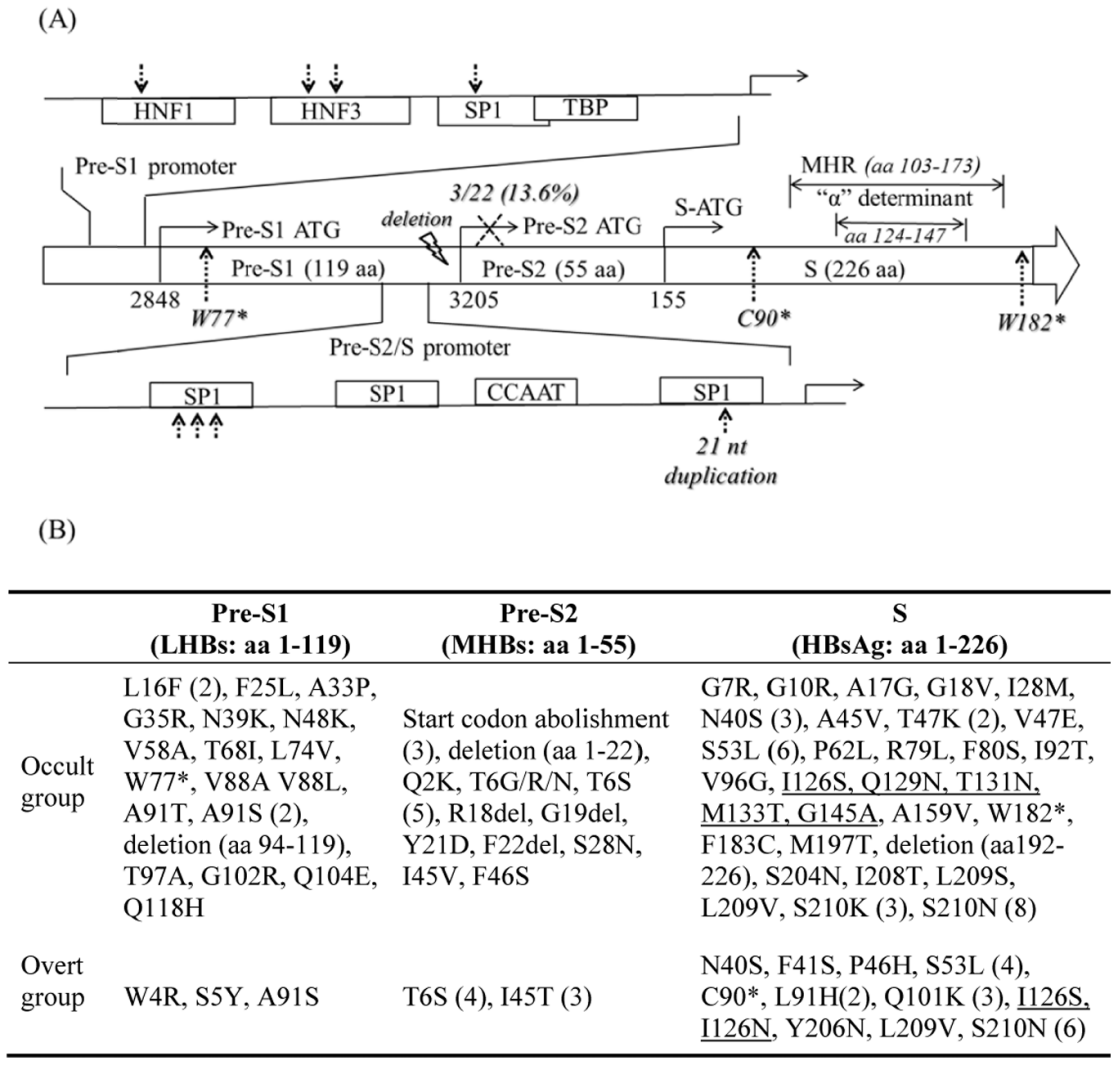
Schematic representation showing types and positions of mutations affecting the HBV pre-S/S region. (A) Binding sites in the pre-S1 and pre-S2/S promoters for the liver-specific transcription factors (HNF1 and HNF3), Sp1, TATA binding protein (TBP) and the CCAAT box. Distinct mutations in TF binding sites (broken arrows), premature termination of surface protein synthesis (*), nucleotide duplication (broken arrows), deletion (a flash) and abolishment of pre-S2/S start codon (X) were indicated. (B) Amino acid changes in the pre-S1, pre-S2 and S genes. (Position with more than one amino acid changes was bracketed. Amino acid changes in “α” determinant region were underlined).

Details of the mutations observed in subjects with OBI and CHB infection were illustrated in Table S2. The total number of amino acid variations over the entire PreS/S region was significantly higher in OBI than control cases [89/400 (22.2%) vs. 33/400 (8.25%), p<0.0001] (Figure 2). The number of amino acid substitutions in individual pre-S1, pre-S2 and S regions were also significantly higher in OBI than control cases [pre-S1∶21/119 (17.6%) vs. 3/119 (2.5%), p<0.0001; pre-S2∶20/55 (36.4%) vs. 7/55 (12.7%), p<0.001; S: 48/226 (21.2%) vs. 23/226 (10.2%), p<0.001], respectively (Figure 2B). Some mutation patterns were uniquely observed in OBI cases in pre-S/S ORF, including sequence deletions (3/22, 13.6%) and abolishment of the pre-S2 start codon (ATG) by a point mutation (3/22, 13.6%). The analysis of S ORF showed that the clinically important amino acid substitutions were mainly located in the major hydrophilic region (MHR) (residues 103–173). These included I126S, I126N, Q129N, T131N, M133T, G145A and A159V. Except for A159V, all the other 6 amino acid substitutions in MHR reside in the “α” determinant region (residues 124–147) of the HBsAg, and were found more frequently in OBI than control cases [5/24 (20.8%) vs. 2/24 (8.3%), p = 0.416]. Among them, I126S, Q129N and M133T are associated with diagnostic problems [15], [31], [32], whereas G145A is known as vaccine escape mutant and associated with OBI [33].

### Mutational Analysis on the pre-C/C Coding Regions

**Figure 3:**
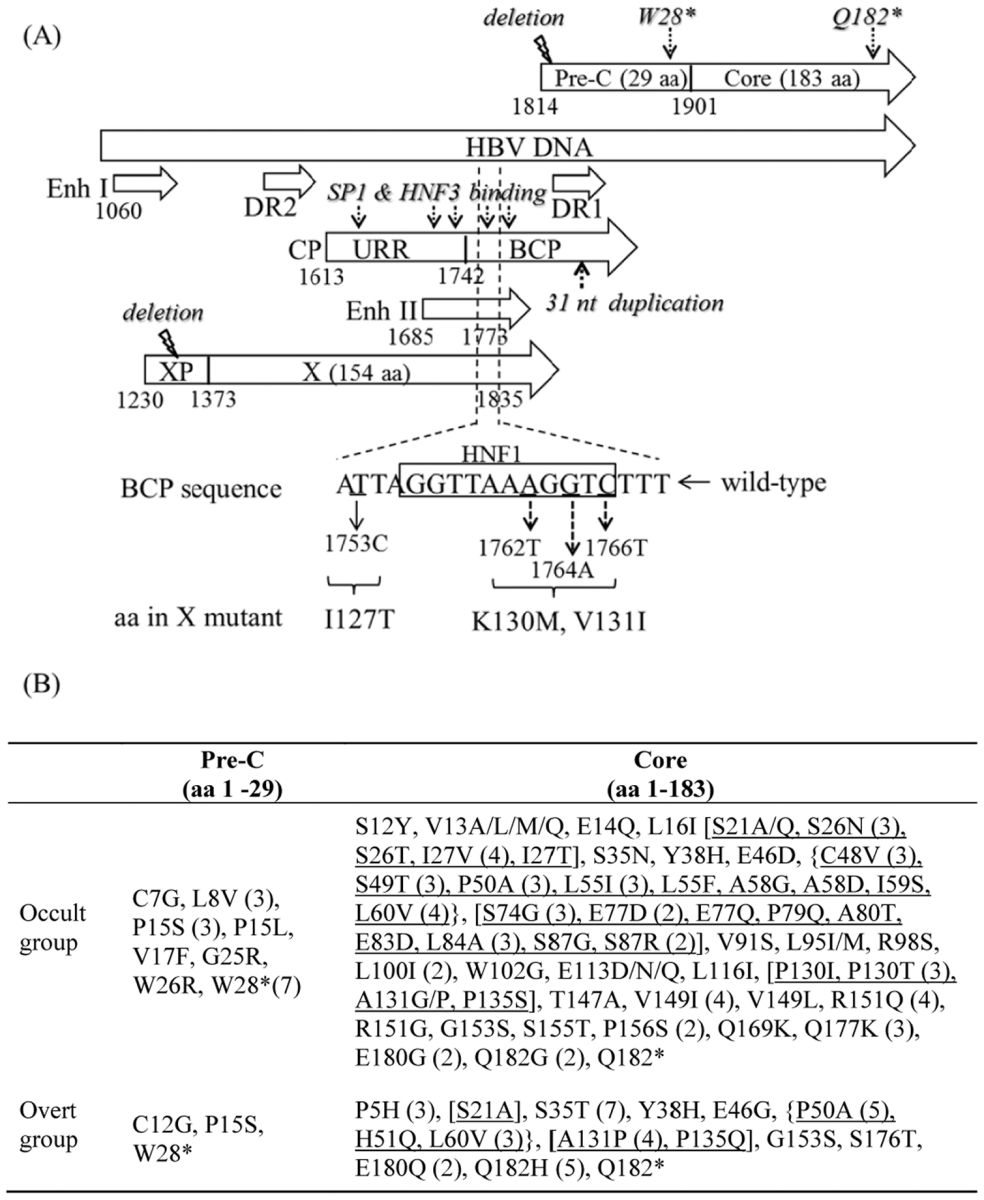
Schematic diagrams showing types and positions of the mutations affecting the overlapping HBV key regulatory regions. (A) The overlapping regions of HBV genome contain core promoter (CP), basal core promoter (BCP), enhancer II (Enh II), DR1 (nt 1824–1834) and DR2 (nt 1590–1600) and coding regions of preC/C and X proteins. Clinically important mutations in BCP that results in the alteration of HNF1 binding site and the corresponding amino acid changes in the overlapping X gene. (aa: amino acid, nt: nucleotide, DR: direct repeat). Distinct mutations in TF binding sites, premature termination of protein synthesis, nucleotide duplication and deletion were indicated. Percentage of X mutants in overlapping regions in occult and control cases were listed. (B) Amino acid changes in the pre-C and core genes. (Position with more than one aa changes was bracketed).

The number of amino acid substitutions in OBI cases was significantly higher than control cases in both pre-C and core ORFs (pre-C: 18/29 (62.1%) vs. 3/29 (10.3%); core: 99/183 (54.1%) vs. 37/183 (20.2%), both with p<0.0001) (Figure 3A, 3B and Table S2). Within the pre-C region, the G1896A mutation introducing a stop signal at codon 28 (W28*), which abolishes HBeAg production was found in 7/22 occult cases and 1/11 control cases (31.8% vs. 9.1%, p = 0.2176) (Figure 3B). Mutations in core region were mainly presented within different antigen immunogenic epitopes. As shown in Figure 3B, OBI cases had a higher number of amino acid substitutions than control cases within the CD8+ CTL-epitope (residues 18–27) [6/10 (60%) vs. 1/10 (10%), p = 0.0573] and CD4+ T-cell epitope (residues 48–69) [9/22 (40.9%) vs. 3/22 (13.6%), p = 0.0883]. Similarly, multiple amino acid substitutions were also detected within the HBcAg domain (residues 80–120). In particular, 9 amino acid changes (S74G, E77D, E77Q, P79Q, A80T, E83D, L84A, S87G and S87R) within the HBc/e1 epitope (residues 75–90) were solely detected in OBI cases (56.3% vs. 0%, p<0.015), and that was known to reduce both HBe and HBc antigenicity [34]. In addition, amino acid substitutions within the HBc/e2 epitope (residues 120–140) in OBI cases were also higher than controls [5/21 (23.8%) vs. 2/21 (9.52%), p<0.041].

### Mutational Analysis on the X Coding Region

The total number of amino acid substitutions in the X ORF was significantly higher in subjects with OBI than controls [83/154 (53.9%) vs. 56/154 (36.4%), p<0.005]. A number of amino acid mutations were solely detected in subjects with OBI, including G22S (13.6%), P38S (13.6%), A102T (13.6%) and K118T (13.6%). There was a trend of a higher frequency of H86R mutation detected in OBI than in control cases [12/22 (54.5%) vs. 2/11 (9.1%), p = 0.067].

### Mutational Analysis on the P Coding Region

The total number of amino acid substitutions over the P ORF was significantly higher in OBI than control cases [197/843 (30.1%) vs. 63/843 (7.6%), p<0.0001] (Table S2). Deletions in the pre-S/S region which lead to deletions and truncated proteins in the overlapping P coding region were found in 3/22 (13.6%) OBI cases. 3 out of 6 (50%) mutations detected in “α” determinant region of HBsAg also caused mutation in overlapping rt region, these included sI126S to rtD134E, sQ129N to rtS137Q and sT131N to rtN139K (Figure 2B). Nucleotide T128A mutation in the finger domain of the rt was uniquely detected in 3/22 (13.6%) OBI cases. It is speculated that rtT128A mutation might result in defective replication activity [35].

### Distinct Mutations in the Regulatory Regions

The mutation frequency within the HBV key regulatory regions was comparable between the OBI group and control group (Table 1). However, a significant difference in the nucleotide diversity was noticed over the pre-S2/S promoter when comparing the OBI with the control groups with genotype C (p = 0.037; Table 2). Point mutations were the most noticeable changes that scattered over these key regulatory elements. Most of them result in interference with liver specific transcription factor (TF) binding sites and were more frequently detected in OBI cases than controls (Figure 2A, Figure 3A and Table S3). Large deletions at nt position 3127–55, 728–1255 and 1754–1771 were found in 3/22 (13.6%) OBI cases. These deletions led to loss of pre-S2/S and X promoters and disruption of BCP promoters, respectively. It is noteworthy that unique nucleotide duplications at the promoter regions were observed in 2/22 (9.1%) OBI cases. These unique nucleotide duplications included a 21 nt duplication (nt 3107–3127) in the Sp1 binding sites within the pre-S2/S promoter and a 31 nt duplication (nt 1644–1674) that interrupted the essential element box β in enhancer II (Table S3) [36].

**Figure 4:**
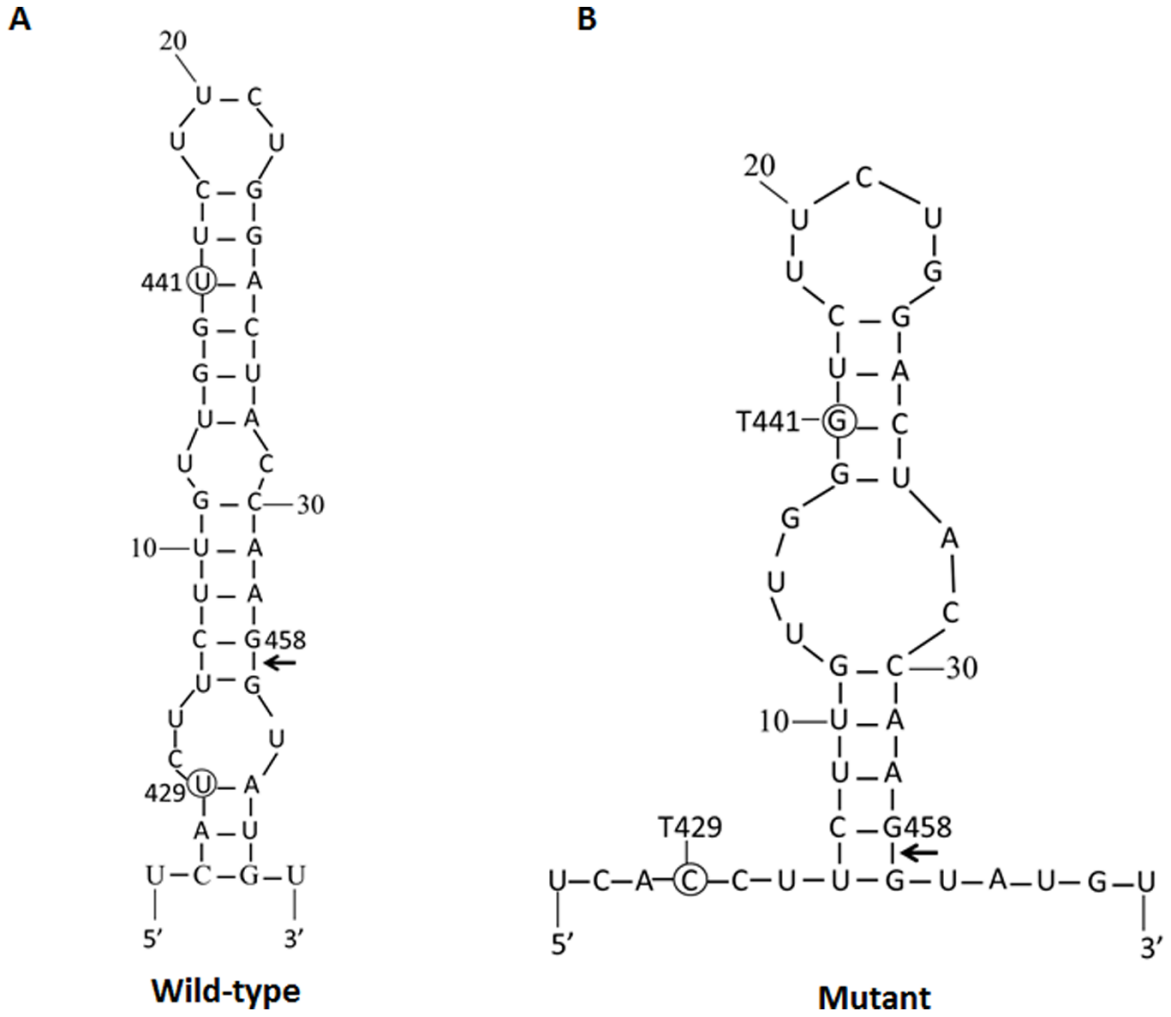
Prediction of RNA secondary structure at the 5′ splice site of occult hepatitis B virus infection (OBI) and control HBV strain. (A) RNA structure around the 5′ splice site at position 458 in control wild-type HBV strain (B) Predicted RNA structure of the OBI case with nucleotide T429C and T441G mutations. (Mutations are encircled and an arrow indicates the splice site).

Recent studies suggested that pre-S2/S mRNA splicing is essential for HBsAg expression [20], [22]. This RNA splicing event is controlled by a 5′ splice donor site (nt 426–464) and the post-transcriptional element (PRE) that contains the 3′ splice acceptor site. Nucleotide G458A mutation was found to influence HBV RNA splicing and HBsAg production [20]. No G458A mutation was detected in this study. One OBI case with both T429C and T441G mutations in the 5′ splice donor site, which were predicted to affect mRNA secondary structure by MFOLD (Figure 4) [20]. The influence of RNA secondary structure on the activity of a 5′splice site of the S mRNA and optimal production of HBsAg needs further functional analysis.

### Functional Analysis of the Distinct Nucleotide Alterations in Regulatory Elements

**Figure 5:**
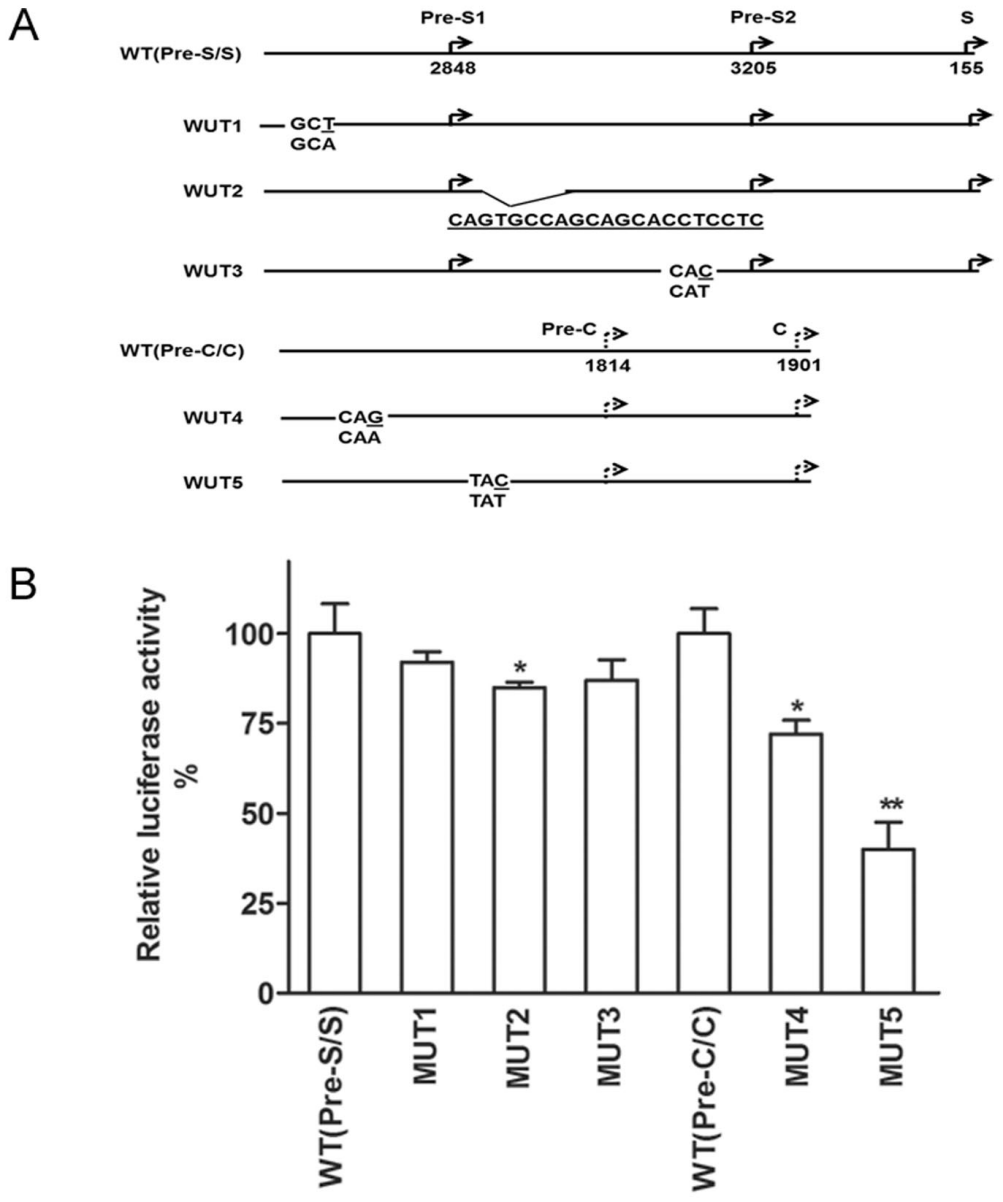
The relative luciferase activities of the constructs were determined in Huh-7 cells. (A) The HBV sequences showing type and position of the mutations in the constructs. (B) The relative luciferase activity in the mutant constructs. Error bars indicate the standard deviations. The luciferase activity of the wild-type (WT) was set to 100% and the changes with the different mutants were calculated accordingly.

We next explored whether the distinct surface promoter and core promoter (CP) mutations identified in OBI cases caused a change in their level of transcription activity. Based on the findings of the mutations in the regulatory regions in OBI cases, 5 mutants and two wild-type controls were constructed. These included T2768A mutation located in Sp1 binding sites of pre-S1 (MUT1), 21 nt duplication between 3128–3148 on pre-S2/S (MUT2), C3015T mutation on preS2/S (MUT3) and two mutations G1677A (MUT4) and C1706T (MUT5) in CP. Luciferase activities of the plasmids containing these mutant promoters (MUT1–5) were compared to that of their corresponding wild-type promoter. The absolute luciferase activity of the control wild-type promoter was set to 100% and all the other mutant constructs were compared accordingly. As shown in Figure 5, mutation significantly affected the promoter activity in MUT2 (85%, p<0.05), but not in MUT1 (92%, p = 0.226) and MUT3 (87%, p = 0.156). The promoter activity was significantly decreased in MUT4 (72%, p<0.05) and MUT5 (40%, p<0.005) with respective to the control CP.

## Discussion

We performed mutational analysis of the RCA-amplified full-length HBV genomes from OBI and control cases. In order to exclude natural polymorphism and/or differences related to the geographical origin of the patients, it is imperative to have a robust comparison of sequences obtained from cases with the same genotype and origin. In this sense, we compared OBI sequences with both genotype-matched controls and reference sequences obtained from 49 genotype C (39 with sub-genotype C1 and 10 with sub-genotype C2) [26] and 5 genotype B2 CHB patients from Hong Kong [30].

Our full-length HBV sequence analysis revealed that HBV nucleotide diversity in OBI cases was significantly higher than control cases, indicating that more sequence variations were observed in OBI. Despite the strict criteria, a variety of mutations have been detected which are scattered throughout the viral genome in both occult and overt cases. Many of the mutations identified were in the surface proteins (Figure 2), which affect HBsAg detection assays, immune response recognition, HBV infectivity and virion morphogenesis [15], [20], [23]. However, we did not identify any single prevailing mutation or genetic signature which was associated with OBI. In this study, a mixture of mutation patterns like point mutations, deletions and nucleotide duplications were identified in OBI cases. This finding of multiple mutations, rather than prevailing mutations or genetic signatures, in OBI cases, is consistent with other reports [15], [16], [20], [21], [23], [37]. This is further confirmed by our mutation frequency analysis that the amino acid mutation frequency within the individual genes and regulatory regions in the OBI cases was higher than that in the control cases. Taken together, it is likely that OBI is attributed to an increased accumulation of mutations in multiple regions, including key regulatory and coding regions, which in turn disrupts HBV replication and gene expression.

Despite the lack of prevailing OBI-associated mutations, this study identified several unique mutations within the HBV regulatory regions that may affect HBV replication. Many of these mutations identified in the key regulatory regions reside in the TF binding sites, which may disrupt TF bindings and hence affect promoter functions (Figure 2*A* and 3*A*). The reduced promoter activity was demonstrated in vitro by our luciferase assay, which showed that core promoter activity was reduced with G1677A mutation in TF HNF3 binding sites and with C1706T mutation in the enhancer II region. We have also identified 2 OBI cases with duplications of HBV regulatory sequence. To our knowledge, this is the first description of nucleotide duplication in regulatory elements in OBI. This may explain the significant decrease in the pre-S2/S promoter activity caused by a 21 nt duplication between 3128–3148 by the luciferase assay.

This study also identified mutations in the “α” determinant region of the preS/S ORF in OBI subjects. Many mutations identified in the pre-S/S ORF, especially within the “α” determinant, have been demonstrated to affect the antigenicity and production of HBsAg, and thus possibly contribute to OBI [20], [23], [38], [39]. This study identified an OBI case with the sG145A mutation, a vaccine escape variant which has been associated with OBI [33]. In addition, point mutations leading to the abolishment of pre-S2/S start codon and early stop signals, and sequence deletions resulting in truncated surface protein synthesis were also found in OBI cases. Xu et al. first reported that expression of a pre-S1 mutant would result in intracellular retention of HBsAg and that an appropriate balance of the various HBV surface proteins was important for functional HBsAg production and secretion [40], [41]. Therefore, it is possible that the novel mutation patterns identified in the present study may disrupt surface protein synthesis and secretion. Of note, the change from sW182 to a stop codon (W182*) was identified in an occult case, which was recently suggested to be associated with OBI [42].

Due to the overlapping arrangement of HBV ORFs, mutations in the pre-S/S region could lead to amino acid changes in the overlapping *P* gene, especially in reverse transcriptase (rt). In particular, three immune escape mutations identified in “α” determinant region in OBI cases also caused mutations in the overlapping rt functional domains, including sI126S → rtD134E, sQ129N → rtS137Q and sT131N → rtN139K. The effect of these rt mutations on HBV replication level remains to be proven by further in vitro analysis.

Mutations associated with post-transcriptional regulation of HBsAg production were detected in OBI cases. The mechanism for the newly identified post-transcriptional regulation of HBsAg expression is still not yet fully elucidated. We have identified one occult case with T429C and T441G mutation at the 5′ donor splice site, which may affect the preS/S RNA secondary structure. As inhibition of HBsAg production could result from the mutation occurred in the pre-S2/S splicing sites [20], [22], [43], further investigation in larger OBI samples are needed.

In conclusion, our results indicated that subjects with OBI had a higher genetic diversity and higher mutation frequency than control subjects. The mutations identified in OBI cases included point mutations and sequence deletions, which might cause premature gene termination, defective HBsAg synthesis, decreased HBV promoter activity, or reduced HBV replication. All these mutations constrain viral replication capacity, and may collectively contribute to the OBI entity among the HBsAg-negative subjects. It is likely that multiple mechanisms are involved, and a combination of mutations, rather than any prevailing genetic signatures, is responsible for the OBI status.

## Supporting Information

Figure S1 **Phylogenetic analysis using the entire HBV genomes amplified from the 22 occult and 11 control HBV infected subjects.** The filled circles represent the occult subjects, and the empty circles represent control subjects. Phylogenetic comparison was done by neighbor-joining algorithm based on Kimura two-parameter distance estimation. Bootstrap values more than 75% are indicated on the major nodes. The scale of the evolutionary distances is shown at the bottom (scale bar). References sequences retrieved from GenBank are indicated by their accession numbers.
(DOCX)

Table S1 **HBV-specific primers for rolling circle amplification (RCA), PCR amplification of full-length HBV genomes, sequencing and regulatory elements analysis.**
(DOCX)

Table S2 **Amino acid changes in HBV coding genes from subjects with occult and overt HBV infections.**
(DOCX)

Table S3 **Nucleotide changes in key regulatory regions of the hepatitis B virus (HBV) genome.**
(DOCX)
