# Supplementary material for: Sequence Variations of Full-Length Hepatitis B Virus Genomes in Chinese Patients with HBsAg-Negative Hepatitis B Infection
Source: PLoS One. 2014 Jun 5;9(6):e99028. doi: 10.1371/journal.pone.0099028 (PMC4047052; doi:10.1371/journal.pone.0099028)
Supplement: Figure S1 — Phylogenetic analysis using the entire HBV genomes amplified from the 22 occult and 11 control HBV infected subjects. The filled circles represent the occult subjects, and the empty circles represent control subjects. Phylogenetic comparison was done by neighbor-joining algorithm based on Kimura two-parameter distance estimation. Bootstrap values more than 75% are indicated on the major nodes. The scale of the evolutionary distances is shown at the bottom (scale bar). References sequences retrieved from GenBank are indicated by their accession numbers. (DOCX) [file pone.0099028.s001.docx]

**Figure S1**

Phylogenetic analysis using the entire HBV genomes amplified from the 22 occult and 11 control HBV infected subjects. The filled circles represent the occult subjects, and the empty circles represent control subjects. Phylogenetic comparison was done by neighbor-joining algorithm based on Kimura two-parameter distance estimation. Bootstrap values more than 75% are indicated on the major nodes. The scale of the evolutionary distances is shown at the bottom (scale bar). References sequences retrieved from GenBank are indicated by their accession numbers.
