# Supplementary material for: Sequence Variations of Full-Length Hepatitis B Virus Genomes in Chinese Patients with HBsAg-Negative Hepatitis B Infection
Source: PLoS One. 2014 Jun 5;9(6):e99028. doi: 10.1371/journal.pone.0099028 (PMC4047052; doi:10.1371/journal.pone.0099028)
Supplement: Table S1 — HBV-specific primers for rolling circle amplification (RCA), PCR amplification of full-length HBV genomes, sequencing and regulatory elements analysis. (DOCX) [file pone.0099028.s002.docx]

**Table S1**. HBV-specific primers for rolling circle amplification (RCA), PCR amplification of full-length HBV genomes, sequencing and regulatory elements analysis

| **Primer** | **^a^Sequence (5' - 3')** | **Nucleotide position^b^** |
| --- | --- | --- |
| ***Primers for RCA*** | |  |
| RCA 1 | AATCCTCACAATA*C*C | 226-240 |
| RCA 2 | GATGGGATGGGAA*T*A | 615-601 |
| RCA 3 | CCTATGGGAGTGG*G*C | 637-651 |
| RCA 4 | GCAACGGGGTAAA*G*G | 1154-1140 |
| RCA 5 | ATGCAACTTTTTC*A*C | 1814-1828 |
| RCA 6 | TCCAAATTCTTTA*T*A | 1930-1916 |
| RCA 7 | TAGAAGAAGAACT*C*C | 2368-2382 |
| RCA 8 | AGAATATGGTGAC*C*C | 2828-2814 |
| ***Primers for full-length HBV amplification*** | | |
| P1 | ccggaaagcttgagctcttcTTTTTCACCTCTGCCTAATCA | 1821-1841 |
| P2 | ccggaaagcttgagctcttcAAAAAGTTGCATGGTGCTGG | 1825-1806 |
| ***Primers for sequencing*** | | |
| 127s | CTCGAGGACTGGGGACCCTG | 127-146 |
| 406s | CTTCATCCTGCTGCTATGCCT | 406-426 |
| 811s | CTTTTGTCTTTGGGTATACAT | 811-831 |
| 1100s | CGCCAACTTACAAGGCCTTTCT | 1100-1121 |
| 2385a | GAGGGAGTTCTTCTTCTAGG | 2385-2366 |
| 2048s | CCTCACCATACTGCACTCA | 2048-2066 |
| 2417s | CGCGTCGCAGAAGATCTCA | 2417-2430 |
| 237a | CGAGTCTAGACTCTGTGGTA | 256-237 |
| ***Primers for S promoters and Core promoter*** | | |
| CP | F: cgaggtaccCTCATCTGCCGGACCGTGTG  R: ctagctagcCAGAGGTGAAAAAAGTTGCA | 1562-1722  1834-1812 |
| SP1 | F: cgaggtaccCGCGTCGCAGAAGATCTCA  R: ctagctagcCTTGTTCCCAAGAATATGGTGA | 2412-2430  2838-2816 |
| SP2 | F: cgaggtaccTCACCATATTCTTGGGAACAA  R: ctagctagcGACTGTCTCTTAGAGGAGGA | 1817-2837  3193-3173 |

Note: HBV sequences are in upper-case letter; the cloning tails are in lower-case letters. Restriction enzyme cutting sites were underlined. *indicates phosphorothioate modifications. Nucleotides use the genotype C numbering system.
