# Supplementary material for: Sequence Variations of Full-Length Hepatitis B Virus Genomes in Chinese Patients with HBsAg-Negative Hepatitis B Infection
Source: PLoS One. 2014 Jun 5;9(6):e99028. doi: 10.1371/journal.pone.0099028 (PMC4047052; doi:10.1371/journal.pone.0099028)
Supplement: Table S2 — Amino acid changes in HBV coding genes from subjects with occult and overt HBV infections. (DOCX) [file pone.0099028.s003.docx]

| **Table S2. Amino acid changes in HBV coding genes from subjects with occult and overt HBV infections** | | | | | | | | | | |
| --- | --- | --- | --- | --- | --- | --- | --- | --- | --- | --- |
| **No.** | **Subjects** | | | **Pre-S1** | **Pre-S2** | **S** | **Pre-C** | **Core** | **X** | **Polymerase** |
| **Occult HBV infection (n =22)** | | | | | | | | | | |
| **1** | | Bx8  (KJ410512) | | F25L,  T68I |  |  | W28* | V13L, S26N, C48V, P50A, A58G, L60V, S74G, E77Q, P79Q, L84A, E113Q, Q177K, Q182G | A102T | **(tp**:D16E, N47D, E73N, Q93E)  (**spacer**: L205V, C213S, P227S) (**rt**:N123H, T128A, A223S, Q267L, I269L)  (**rh**:Q23R, R113H) |
| **2** | | Bx9  (KJ410521) | |  |  | I92T, V96G, A45V, L209S | V17F, W28* | I27V, S49T, E77D, S87G, E113D, V149I, R151Q, S155T | R26C, P38S, S101A, V116L, I127T, F132Y | (**tp**:L46P, Q143K)  (**spacer**:P212S)  (**rt**:T7A, T54S, S117P, A223S, N238H, V266I, Q267L)  (**rh**:Q23R, R113H) |
| **3** | | Bx10  (KJ410511) | | G35R, V88A | Start codon abolishment, T6R, F46S | N40S, S53L, Q129N, S210N |  | V13M, P50A, L55I, E83D, L95I, L116I, P130I, V149I, R151Q, Q177K, Q182G | H86R, K130M, V131I | (**tp**:N47D, K55R, V119H)  (**space**r:C213K)  (**rt**:H9Y, R110G, S119A, S137Q, N238T, Q267H, N337D)  (**rh**:D10V) |
| **4** | | Bx76  (KJ410514) | | G102R,  Q104E | Start codon abolishment, T6N | I126S, T131N, M133T, S210N | P15S | S26N, I27V, L84A, S87R, A131G, V149L | H52N, M79V, H86R, H94Y, I127F, K130M, V131I | (**tp**:L13F, R35H, Q85K, V119H) (**spacer**:H129Y, R246G)  (**rt**:D134E, N139K) |
| **5** | | Bx313  (KJ410518) | |  | T6S, I45V | S53L, A159V, S210K |  | E46D, P50A, S74G, P130T, R151G, Q169K | H86R, L98I, I127T, K130M, V131I | (**tp**:D15E, Q85K, V119R)  **(rt**: H9Y, L115V, M129L, L217R, S219A, N238T, E263D, Q267H,V278I) |
| **6** | | Bx315  (KJ410504) | |  | T6S, S28N | S53L, S210K | W28* |  | H86R, I127F | (**tp**:G32D, Q85K)  (**rt**: H9Y, D31N, N238T, Q267H, V278I) |
| **7** | | Bx317  (KJ410509) | | N39K, N48K, L74V, Q118H | Start codon abolishment, T6G | A17G, G18V, I28M, T47K, P62L, Del (192-226) | P15S | V13A, E14Q, L16I, S21A, I27V, L55I, I59S, E77D, A80T, L84A, E113N, R151Q, P156S, E180G | P38S, A47T, K118T, I127V, K130M, V131I | (**tp**:D16N, N45D, L46P E73N, Q93T, C169F)  (**spacer**:H129D, del:202-346, P227S, Q229K, R246G)  **(rt**: H9Y, T37A, del201-374)  (**rh**:del1-32,Y108C) |
| **8** | | Bx318  (KJ410502) | |  |  | N40S, F80S | W28* | S26N, S49T, L60V, P135S, V149I, G153S, Q177K | C6Y, A44L, A47T, K95E, K118T, I127L | (**tp**:D16E, E73N, V101T, N118K, L123F, R164H)  (**rt**:A222T, N238H, C332R,K333Q)  (**rh**:A77T) |
| **9** | | Bx319  (KJ410515) | |  | Q2K, T6S | N40S, T47K, R79L, S204N, S210K | L8I, P15L, G25R, W28* | S12Y, V13Q, S26T, S74G, A131P | R26S, A44T, A47T, R78C, H86R, I127S, K130M, V131I, F132Y | (**spacer**: V185D)  (**rt**: H9Y, R114S, S219T, N238A, Q267H, V278T, A329T, C332R, K333N)  (**rh**:I55L) |
| **10** | | Bx320  (KJ410505) | | T97A | T6S  delR18, delG19, Y21D, delF22 | S53L |  | C48V, L100I, P130T | D48G, A102T | (**tp**:S49D, Q85K, V119R)  (**spacer**:del317-319, V185D)  (**rt**: H9Y, A223S, Q267L, I269L, A329T)  (**rh**:Q23R, R113H, T119A) |
| **11** | | Bx321  (KJ410500) | |  |  | V47E, M197T, I208T, S210K | W28* | S21Q, I27V, S49T, L55I, L60V, V91S, T147A, V149I, R151Q, P156S, E180G | A47T, R72L, H87R, K118T, I127V, K130M, V131I, S144P | (**tp**:N45D, S49N, E73N, C169F)  (**spacer**:K219T)  (**rt**:S219A, A222T N238H)  (**rh**:A143S) |
| **12** | | Bx323  (KJ410510) | | A33P |  | F183C | W26R | S35N, Y38H, L55F, A58D, L95M, R98S, W102G |  | (**tp**:Q143K)  (**spacer**:C213S)  (**rt**:A223S, L229M, Q267L, I269L)  (**rh**:R113H, D138A) |
| **13** | | Bx346  (KJ410508) | |  |  | S210N | L8I | C48V, L100I, P130T | G22S, S42A, D48V, H49N, A102T | (**tp**:Q143K)  (**rt**:N238T, Q267H, V278I, A329T) (**rh**:I55L) |
| **14** | | Bx514  (KJ410519) | |  |  | S210N |  |  | G22S, H86R | (**spacer**:R246G)  (**rt**: H9Y, V278I) |
| **15** | | Bx523  (KJ410513) | |  |  | S53L, S210N |  |  | A85V, H86R | (**tp**:Q85K, V119R)  (**rt**: H9Y, V23I, N238T, Q267H, V278I, K333Q)  (**rh**:I55L) |
| **16** | | Bx526  (KJ410506) | | V88L |  | S210N | P15S | I27T, S87R | G22S, R26C, H86R | (**tp**:Q75K, Q85K)  (**spacer**:R246G)  (**rt**: H9Y, A223T, V278I, K328Q)  (**rh**:S60A) |
| **17** | | Bx532  (KJ410501) | |  | T6S | S53L, S210N |  |  | P38S, H86R | (**tp**:Q85K, V119R)  (**rt**: H9Y, V23I, N238T, Q267H, V278I, K333Q)  (**rh**:I55L) |
| **18** | | Bx534  (KJ410503) | | V58A  W77*, A91T, del (94-119) | Del (1-22) | W182* | W28* | L60V, Q182* | H86R, Del (128-133) | (**tp**:Y116F, Q143K)  (**spacer**:del275-22)  (**rt**:T128A, L132M, V191I, A223S, Q267L, I269L)  (**rh**:Q23R, K66N, R113H) |
| **19** | | OS4777  (KJ410516) | | L16F |  |  |  |  | A44V, A47T, I127V | (**tp**:D16E)  (**spacer**: S196F) |
| **20** | | OS4869  (KJ410517) | | L16F |  |  |  |  | A44V, A47T, I127V | (**tp**:D16E) |
| **21** | | OS4901  (KJ410520) | | A91S |  | G7R | C7G, L8V |  | A44V, A47T, I127V, R72H | (**tp**:E73Q, V101T, Q143K)  (**spacer**:F197V)  (**rt**:R15T, R18K, T128A, A223S, Q267L, I269L)  (**rh**:Q23R, R113H, A143T) |
| **22** | | OS6840  (KJ410507) | | A91S |  | G10R, I68T, G145A |  |  | R72H, H86R | (**tp**:S165I) |
| **Overt HBV infection (n = 11)** | | | | | | | | | | |
| **1** | **C0015**  (KJ410498) | |  | |  | S210N |  | P5H, S35T, P50A, Q182H | T36D, H86C, I127T, K130M, V131I | (**tp**:N47T, Q143K)  (**rt**:P99T, N238T, Q267L) |
| **2** | **C1095**  (KJ410496) | |  | | T6S | S53L, Q101K, I126S, S210N |  |  | T36D, H86C, I127T, K130M, V131I | (**tp**:Q143K)  (**rt**:N238T, Q267H, V278I) (**rh**:I55L) |
| **3** | **C1100**  (KJ410494) | |  | |  |  |  | S35T, H51Q, L60V, E180Q | I127T, K130M, V131I, F132Y | (**tp**:I156V)  (**spacer**:R246G)  (**rh**:S2A, S64A) |
| **4** | **C1202**  (KJ410489) | | S5Y | | T6S | F41S, S53L, Q101K, S210N |  | S35T, P50A, A131P, Q182H | C6D, T36D, H86C, K130M, V131I, I127T | (**tp**:N47T, Q85K, I156V)  (**rt**:S246C, V278I, V278T, N337D)  (**rh**:S2A, S64A, V90A, L107I) |
| **5** | **C1204**  (KJ410493) | |  | | I45T | N40S, S53L, S210N |  | S21A, Y38H, E46G, L60V, Q182* | S42A, H86R, H94Y, I127T, K130M, V131I | (**tp**:L46P, Q85K, I119R)  (**spacer**:F186L, S189A)  (**rt:**Y111H, V214E, Q267H) |
| **6** | **C1313**  (KJ410492) | |  | |  | L91H |  | S35T, P50A, A131P, Q182H | T36D, H86C, K130M, V131I | (**tp**:Q85K, )  (**rt**:V278I )  (**rh**:S64A, L107I) |
| **7** | **C1323**  (KJ410491) | | W4R | | T6S, I45T | T47R, Q101K, I126N, L, 209V, S210N | P15S | S35T, G153S | R26C, V30F, H86R, I127L, I141M, V131I | (**tp**:N47T, Q85K, N118K) (**spacer**:L184S)  (**rt**:I122V, L217R, V278I)  (**rh**:S2A) |
| **8** | **C1324**  (KJ410495) | |  | |  | P46H, S53L, , Y206N, S210N |  | P5H, S35T, P50A, A131P, Q182H | T36D, H86C, I127T, I141M, K130M, V131I | (**tp**:Q85K)  (**rt**:N238T, M250I, Q267H)  (**rh**:S2A, S64A, V90A, L107I) |
| **9** | **C1325**  (KJ410497) | | A91S | |  | C90* | C12G | P135Q, S176T | I127T, K130M, V131I, F132Y | (**tp**:D40E)  (**rt**:Q319L)  (**rh**:H22N) |
| **10** | **C1328**  (KJ410499) | |  | | T6S, I45T |  | W28* | P5H, S35T, P50A, A131P, Q182H | T36D, H86C, I127T, R138K, K130M, V131I | (**tp**:N47T, Q85K)  (**rt**:V278I)  (**rh**:L107I) |
| **11** | **C1329**  (KJ410490) | |  | |  |  |  | L60V, E180Q | A44L, A47T, I127V, K130M | (**tp**:D16E )  **(rt**:Q319L, Q334K) |
