# Supplementary material for: Sequence Variations of Full-Length Hepatitis B Virus Genomes in Chinese Patients with HBsAg-Negative Hepatitis B Infection
Source: PLoS One. 2014 Jun 5;9(6):e99028. doi: 10.1371/journal.pone.0099028 (PMC4047052; doi:10.1371/journal.pone.0099028)
Supplement: Table S3 — Nucleotide changes in key regulatory regions of the hepatitis B virus (HBV) genome. (DOCX) [file pone.0099028.s004.docx]

**Table S3**: Nucleotide changes in key regulatory regions of the hepatitis B virus (HBV) genome

| **HBV region** | **Control group** | **Occult group** |
| --- | --- | --- |
| **Enh I**  (nt 1060-1260) | T1060C (2), A1074T, G1079T (2), C1110T (2), A1138G, T1165G (3), C1191T, T1206C | T1060A, A1068C, A1072G (2), G1078T, C1092A, C1110T (3), A1111C, G1114A (3), C1116A, C1120T, T1123C, A1126C (3), A1128T, C1135T, A1138G, A1167C (2), T1173C, C1191T, C1218T, A/G1221T (2), A1229G, G1230C (3), A1242T, T1254G |
| **Enh II**  (nt 1635-1714) | C1653T, A1679C (3) | 31 nts duplication (1644-1674), C1653T, A1656G, T1674G, G1677A (3), A1679C (3), C1706T (2) |
| **Core promoter**  (nt 1519-1822) | G1613 (2), C1629T (3), A1752C, T1753C (4), A1762T (6), G1764A (6), T1768A (2), T1794A (2) | C1527A, A1574C, A1574T, G1588T (2), C1605T, A1608G, G1613A (8), C1627T (2), C1629T, G1632A, 31 nts duplication (1644-1674), G1719T, A1726C (2), A1727G (3), G1739A, G1742A, A1752C, A1752T (3), A1752G (4), T1753C (2), T1753G, T1754G, deletion (1754-1771), A1762T (6), G1764A (7), C1766T (2), T1768A (2), T1803C |
| **X promoter**  (nt 1230-1374) | A1317G, C1321T, T1323G, C1321T, C1326A, C1326T (3), C1329A, T1351G (3), T1371C (2) | G1230C (3), deletion (1230-1255), A1242T, T1254G, T1272C, C1282T, T1290C, A1306G, A1317G (8), C1321A, T1323G, C1329A, T1339G, C1350T, A1359C, A1368G |
| **Pre-S1 promoter**  (nt 2710-2800) | T2716C (2), A2721G (2), C2733A (2), G2759A, T2771C (2), A2772G (2), T2775C (2) | T2716C (3), A2721G, C2733A (2), T2738A, T2753G (2), T2768A, G2771C, T2771C (2), T2775C (3), G2797A, G2800T (3) |
| **Pre-S2/S promoter**  (nt 2960-3184) | C3008A, A3054C, A3099C (2), A3108C, G3118T (2) | G2962C, C2985T (2), C2991A, C3008A (2), C3015T (3), T3020C, T3026C, A3030G, A3030T, C3050T, T3066C, C3067T, C3067G, T3069C, T3070C, G3075A, G3078A, T3084C, C3091G, 21 nts duplication (nt 3107-3127), G3109T, T3110C, C3116T, G3118T, G3120A, deletion (3127-55) G3151A, C3157G, A3158G, T3172A, C3181A, A3184C |
| **DR1**  (nt 1824-1834) | **-** | T1832G, C1835A |
| **DR2**  (nt 1590-1600) | **-** | **-** |

**Note**: Nucleotide (nt) changes in occult and control samples (number of samples). Changes are in comparison with consensus sequences created from references of the same genotypes from Genebank.
